# A brief report: de novo copy number variants in children with attention deficit hyperactivity disorder

**DOI:** 10.1038/s41398-020-0821-y

**Published:** 2020-05-12

**Authors:** Joanna Martin, Grace Hosking, Megan Wadon, Sharifah Shameem Agha, Kate Langley, Elliott Rees, Michael J. Owen, Michael O’Donovan, George Kirov, Anita Thapar

**Affiliations:** 1 MRC Centre for Neuropsychiatric Genetics and Genomics, Division of Psychological Medicine and Clinical Neurosciences, Cardiff University, Cardiff, UK; 2 Neuroscience and Mental Health Research Institute, Division of Psychological Medicine and Clinical Neurosciences, Cardiff University, Cardiff, UK; 3 Cwm Taf Morgannwg University Health Board Health Board, Wales, UK; 4 School of Psychology, Cardiff University, Cardiff, UK

**Keywords:** Genomics, ADHD

## Abstract

Recent case–control genetic studies of attention deficit hyperactivity disorder (ADHD) have implicated common and rare genetic risk alleles, highlighting the polygenic and complex aetiology of this neurodevelopmental disorder. Studies of other neurodevelopmental disorders, such as autism spectrum disorder (ASD), Tourette disorder, developmental delay/intellectual disability and schizophrenia indicate that identification of specific risk alleles and additional insights into disorder biology can be gained by studying non-inherited de novo variation. In this study, we aimed to identify large de novo copy number variants (CNVs) in children with ADHD. Children with a confirmed diagnosis of ADHD and their parents were genotyped and included in this sample. We used PennCNV to call large (>200 kb) CNVs and identified those calls that were present in the proband and absent in both biological parents. In 305 parent–offspring trios, we detected 14 de novo CNVs in 13 probands, giving a mutation rate of 4.6% and a per individual rate of 4.3%. This rate is higher than published reports in controls and similar to those observed for ASD, schizophrenia and Tourette disorder. We also identified de novo mutations at four genomic loci (15q13.1–13.2 duplication, 16p13.11 duplication, 16p12.2 deletion and 22q11.21 duplication) that have previously been implicated in other neurodevelopmental disorders, two of which (16p13.11 and 22q11.21) have also been implicated in case–control ADHD studies. Our study complements ADHD case–control genomic analyses and demonstrates the need for larger parent–offspring trio genetic studies to gain further insights into the complex aetiology of ADHD.

## Introduction

Attention deficit hyperactivity disorder (ADHD) is a highly heritable (~70%), common and impairing neurodevelopmental disorder with a complex genetic architecture^1^. Recent case–control genome-wide studies point to the involvement of thousands of relatively common single-nucleotide polymorphisms^2^, very rare protein-truncating sequence variants and likely pathogenic missense mutations^3^, as well as large, rare copy number variants (CNVs)^4–6^. Although all of these classes of variant are associated with ADHD risk, robustly implicating specific risk alleles is an ongoing process. For instance, only a handful of CNV loci (e.g., 15q13.3)^5^ have been individually implicated in risk of ADHD and given the large size of these loci, the specific genes involved are yet to be pinpointed. Studies of de novo mutations using parent–offspring trios provide an especially powerful approach to gene discovery because the background rate in unaffected individuals is low, and therefore if an elevated rate in cases is demonstrable, such mutations are likely pathogenic and can provide important insights into disease biology^7^.

De novo likely damaging mutations, that include CNVs which intersect genes, have been identified for other neurodevelopmental disorders, such as autism spectrum disorder (ASD), Tourette disorder, developmental delay/intellectual disability (DD/ID) and schizophrenia^7–12^. Individuals with ASD have a rate of de novo CNVs that is between three- and fivefold higher than in unaffected siblings or control individuals^13^. Individuals with simplex Tourette disorder have a similar rate of de novo CNVs to probands with ASD^14^ as do individuals with schizophrenia^7^. Of these neurodevelopmental disorders, the rate of de novo CNVs is highest in individuals with DD/ID, at ~10%^15^.

Trio-based genetic studies of ADHD are lacking. There has been only one small study and it reported a rate of de novo CNVs (1.7% or three probands out of *N* = 173) that was only slightly elevated above previous reports of de novo rates in controls^16^. In this study, we set out to characterise large de novo CNVs in parent–offspring trios of probands diagnosed clinically with ADHD.

## Method

### Sample

Children with ADHD, aged 5–17 years old, were recruited from UK child and adolescent mental health and paediatric clinics. ADHD diagnoses based on DSM-IV or DSM-III-R were confirmed via a semi-structured research diagnostic interview (the Child and Adolescent Psychiatric Assessment^17^) by trained, supervised psychologists. Full-scale IQ was assessed using the WISC-IV^18^. A proportion of the probands in the sample (*N* = 136 or 44.6%) have been analysed in previous case–control CNV studies^4,5^. Approval for the study was obtained from the North West England and Wales Multicentre Research Ethics Committees. Written informed consent to participate was obtained from parents and children aged 16 years and older and assent was gained from children under 16 years of age.

### Genotyping, CNV calling and quality control (QC)

DNA was obtained from blood and oragene (saliva) samples for ADHD probands and both biological parents. In all, *N* = 424 ADHD parent–offspring trios were genotyped using a custom version of the Illumina PsychChip. Only samples with >95% call rate and those that passed a sex check were retained. Only complete trios that passed QC and an additional Mendel check in PLINK (to confirm biological relatedness) were used for CNV calling.

CNVs were called using PennCNV^19^ and nearby calls were merged if the CNVs were separated by <50% of their combined length. CNVs were removed if they were called using <10 probes, had size <50 kb, probe density <20 kb/probe or a PennCNV confidence score <10. Samples were removed if they had log R ratio standard deviation > 0.3, B allele frequency (BAF)-drift >0.01 or waviness factor > 0.05. In addition, we applied further, more stringent criteria in ADHD probands and excluded individuals with extreme scores on QC metrics (>4 SD than the sample mean) as follows: if they had an apparently excessive CNV load (number of segments > 50.8 or length in kilobases (KB) > 16593) or poor probe variance (BAF-SD > 0.063). These additional steps were performed to ensure that potential de novo CNVs would be of high quality, whereas being conservative and not removing a whole trio based on reduced data quality in just one parent. In total, *N* = 305 complete trios passed all of the above QC and were kept for analyses. After these steps, only CNVs of size >200 kb were retained for analysis, to ensure accuracy of calls.

CNVs were then annotated as “transmitted”, “non-transmitted”, or “likely de novo” based on presence or absence of a call made in either parent and/or proband. All CNVs that were flagged as likely de novo were visually examined in the offspring and both confirmed biological parents and only clear-cut, un-ambiguous de novo calls were retained for analyses, minimising the false positive rate at the expense of potentially missing real calls. We did not perform validation, as the traces were definitive (see Supplementary Figures 1A–G).

We examined whether the loci disrupted by the de novo CNVs had previously been implicated in case–control common variant^2^ and rare CNV studies^4–6^ of ADHD. Next we compared the list of de novo loci with rare CNV loci implicated in other neurodevelopmental disorders, using previously published studies of ASD^8^, DD/ID^20^, schizophrenia^21,22^, and Tourette disorder^14^. We also annotated each de novo CNV locus with genes and examined if any of these genes have been implicated in ASD, ADHD or DD/ID, based on studies of loss-of-function exonic mutations and deleterious missense variation^3,9,23^. A number of regions and genes overlapped across disorders. The merged lists of neurodevelopmental genetic risks consisted of 96 unique CNV loci and 197 unique genes. All loci were converted to genome build hg19. Overlap was defined as a CNV overlapping at least 50% of a previously implicated region or a CNV affecting the coding sequence of a gene in the list of genes of interest.

## Results

In the *N* = 305 trios (of which, 36 or 11.8% were female probands) that passed QC, we detected a total of 14 de novo CNVs (*N* = 9 that were >500 kb and *N* = 5 that were between 200 and 500 kb) in 13 ADHD probands, with one individual having two CNVs that were >500 kb. Two of the de novo CNV carriers were female and the rest were male. The overall mutation rate was 4.6% (14/305) and the per individual rate was 4.3% (13/305). Although we did not have population controls available in this study, a recent study of control trios using similar methodology reported a de novo mutation rate of 1.0% for CNVs >200kb^24^; thus the rate in ADHD was over four times higher than this comparable control rate. The mutation rate of de novo deletions was 3.3% and the rate of de novo duplications was 1.3%.

The mean IQ of de novo CNV carriers was 83.6 (SD = 13.0) and the mean IQ of the rest of the sample was 85.2 (SD = 13.5), with 3 (25.0%) of the de novo CNV carriers (with non-missing IQ) having comorbid ID (IQ < 70), and 26 (9.6%) of the rest of the sample having comorbid ID. The group of de novo CNV carriers was too small to perform a statistical comparison. Information on age of the parents at birth of their children was available for only 10 of the de novo CNV carriers (maternal age mean(SD) = 24.5(3.4); paternal age mean (SD) = 27.1(5.8)) and the means of the rest of the samples were: maternal age mean(SD) = 26.9(5.8); paternal age mean(SD) = 29.8(6.8). As with IQ, the sample was too small for a robust statistical comparison.

Table 1 summarises the identified CNVs, annotated with affected genes. None of these genes overlapped with genes implicated by a recent ADHD genome-wide association study (GWAS)^2^ or the list of genes implicated by recent sequencing studies of ASD, ADHD and DD/ID^3,9,23^. However, four of the de novo CNVs have previously been robustly implicated in other neurodevelopmental disorders^8,14,20–22^, two of which have also been implicated previously in case–control CNV studies of ADHD^4–6^, as summarised in Table 2. In addition, duplications at 16p13.11^25^ and a de novo duplication at 15q13.1^26^ have also previously been observed in people with schizophrenia. Given that a proportion of the current sample have previously been included in published case–control CNV studies^4,5^, we determined that eight of the de novo loci in our study overlap at least partially (≥50% overlap) with a previously reported CNV in a person with ADHD, with six of these eight de novo CNV carriers having taken part in the previous case–control studies. On the other hand, six of the loci in our study (4p16.3, 10q11.22q11.23, 10q21.3, 10q22.2, 15q26.3 and 16p13.3) are in novel regions and have not been previously reported in individuals diagnosed with ADHD in these large case–control studies.

**Table 1:** A summary of the identified de novo CNVs.

Chr	Start	End	Locus	Type	PennCNV score	No. of probes	Size	No. genes hit	Genes
4	71566	1818625	4p16.3	DEL	234.044	654	1747059	36	ZNF595, ZNF718, ZNF876P, ZNF732, ZNF141, ABCA11P, ZNF721, PIGG, PDE6B, ATP5I, MYL5, MFSD7, PCGF3, LOC100129917, CPLX1, GAK, TMEM175, DGKQ, SLC26A1, IDUA, FGFRL1, RNF212, TMED11P, SPON2, LOC100130872, CTBP1, CTBP1-AS1, MAEA, UVSSA, CRIPAK, FAM53A, SLBP, TMEM129, TACC3, FGFR3, LETM1
4*	66110165	66881057	4q13.1–13.2	DEL	379.845	123	770892	2	EPHA5, LOC100144602
4*	89020503	90256211	4q22.1	DUP	547.522	352	1235708	11	ABCG2, PPM1K, HERC6, HERC5, PIGY, HERC3, NAP1L5, FAM13A-AS1, FAM13A, TIGD2, GPRIN3
10	48302618	51832220	10q11.22–11.23	DEL	1076.37	738	3529602	42	ZNF488, RBP3, GDF2, GDF10, PTPN20A, PTPN20B, FRMPD2P1, BMS1P1, BMS1P5, GLUD1P7, FAM25B, FAM25C, FAM25G, LOC399753, FRMPD2, MAPK8, ARHGAP22, WDFY4, LRRC18, MIR4294, VSTM4, LOC100506733, FAM170B, C10orf128, C10orf71, DRGX, ERCC6, PGBD3, CHAT, SLC18A3, C10orf53, OGDHL, PARG, AGAP8, LOC728407, AGAP7, MSMB, NCOA4, TIMM23, AGAP6, FLJ31813, FAM21A
10	68626126	68875634	10q21.3	DEL	110.26	39	249508	1	LRRTM3
10	75940906	76360232	10q22.2	DEL	19.325	22	419326	1	ADK
11	5653807	7482226	11p15.4	DEL	817.108	1198	1828419	52	TRIM34, TRIM6-TRIM34, TRIM5, TRIM22, OR56B1, OR52N4, OR52N5, OR52N1, OR52N2, OR52E6, OR52E8, OR52E4, OR56A3, OR56A5, OR52L1, OR56A4, OR56A1, OR56B4, OR52B2, OR52W1, C11orf42, FAM160A2, CNGA4, CCKBR, PRKCDBP, SMPD1, APBB1, HPX, TRIM3, ARFIP2, FXC1, DNHD1, RRP8, ILK, TAF10, TPP1, DCHS1, MRPL17, GVINP1, OR2AG2, OR2AG1, OR6A2, OR10A5, OR10A2, OR10A4, OR2D2, OR2D3, ZNF215, ZNF214, NLRP14, RBMXL2, SYT9
15	28962131	30369914	15q13.1–13.2	DUP	295.843	234	1407783	8	WHAMMP2, LOC100289656, LOC646278, GOLGA6L7P, APBA2, FAM189A1, NDNL2, TJP1
15**	102165387	102398631	15q26.3	DEL	188.834	87	233244	5	TM2D3, TARSL2, OR4F6, OR4F15, OR4F13P
16	6186577	6462231	16p13.3	DEL	186.718	50	275654	1	RBFOX1
16	15493046	16251647	16p13.11	DUP	85.574	266	758601	9	MPV17L, C16orf45, KIAA0430, NDE1, MIR484, MYH11, FOPNL, ABCC1, ABCC6
16	21966869	22392905	16p12.2	DEL	181.716	144	426036	7	UQCRC2, PDZD9, C16orf52, VWA3A, EEF2K, POLR3E, CDR2
20	3090124	3891896	20p13	DEL	1115.83	407	801772	20	UBOX5, UBOX5-AS1, FASTKD5, PROSAPIP1, DDRGK1, ITPA, SLC4A11, C20orf194, ATRN, GFRA4, ADAM33, SIGLEC1, HSPA12B, C20orf27, SPEF1, CENPB, CDC25B, AP5S1, MAVS, PANK2
22	18892575	21460220	22q11.21	DUP	129.788	824	2567645	67	DGCR6, PRODH, DGCR5, DGCR9, DGCR10, DGCR2, DGCR11, DGCR14, TSSK2, GSC2, SLC25A1, CLTCL1, HIRA, MRPL40, C22orf39, UFD1L, CDC45, CLDN5, LOC150185, Sep-05, SEPT5-GP1BB, GP1BB, TBX1, GNB1L, C22orf29, TXNRD2, COMT, MIR4761, ARVCF, C22orf25, MIR185, DGCR8, MIR3618, MIR1306, TRMT2A, RANBP1, ZDHHC8, LOC388849, LOC284865, LOC150197, RTN4R, MIR1286, DGCR6L, LOC729444, TMEM191B, PI4KAP1, RIMBP3, ZNF74, SCARF2, KLHL22, MED15, POM121L4P, TMEM191A, PI4KA, SERPIND1, SNAP29, CRKL, AIFM3, LZTR1, THAP7, THAP7-AS1, TUBA3FP, P2RX6, SLC7A4, P2RX6P, LOC400891, BCRP2

All coordinates are genome build hg19.

* These two CNVs were located in the same individual; *DEL* deletion, *DUP* duplication.

** This individual has been independently diagnosed with ring chromosome 15 by a clinical geneticist.

**Table 2:** Identified de novo CNV regions that have previously been robustly implicated in ADHD and other neurodevelopmental disorders.

CNV	CNV locus previously implicated in ADHD or another disorder				
	ADHD	ASD	DD/ID	SCZ	TD
15:28962131–30369914 (DUP)					DUP
16:15493046–16251647 (DUP)	DUP		DEL/DUP	DUP	
16:21966869–22392905 (DEL)			DEL	DEL	
22:18892575–21460220 (DUP)	DEL/DUP	DEL/DUP	DEL/DUP	DEL	DUP

*ASD* autism spectrum disorder, *DD/ID* developmental delay/intellectual disability, *SCZ* schizophrenia, *TD* Tourette disorder.

Of the samples included in previously published studies^4,5^, six were de novo CNV carriers, giving a de novo CNV rate of 4.4%. Of the samples not previously published, six were de novo CNV carriers, giving a comparable de novo CNV rate of 4.1%.

## Discussion

In this study, we present observations on de novo CNVs from the largest published trio-based study of ADHD to date. The overall mutation rate for de novo CNV carriers was 4.6%, which is a similar rate to that observed for two other DSM-5 childhood neurodevelopmental disorders: ASD and Tourette disorder^13,14^, and somewhat higher than observed for schizophrenia^7^. Notably, the rate we observed is substantially higher than that which has been reported for controls^24^ and the previous and only trio-based study of ADHD that observed an overall rate of 1.7%^16^. Aside from the lower power of this previous, smaller study, one potential explanation for the difference is that, unlike Lionel and colleagues^16^, we did not exclude those with an IQ < 70. However, in the current study, only three of the 13 individuals who were carrying de novo CNVs had an estimated IQ < 70, with one further individual who had missing IQ data. The de novo CNV rate in children with ADHD without ID (IQ > 70) was 3.5% in our study, compared to the 1.7% rate previously observed^16^.

The identified CNVs affected a total of 262 genes. None of the genes spanned by the de novo CNVs have been highlighted by the only large exome sequencing study of ADHD to date^3^ or implicated in ADHD based on a recent, large case–control GWAS^2^. However, several of the CNVs have been implicated by previously published ADHD case–control CNV association studies (which will include inherited and de novo CNVs); these include the robustly implicated 16p13.11 duplication and the 22q11.2 duplication^4–6^.

The overlap of de novo CNV loci in the regions 15q13.1–13.2, 16p13.11, 16p12.2 and 22q11.21 with loci previously implicated in ASD, DD/ID, Tourette disorder and schizophrenia is consistent with the notion that neuropsychiatric CNVs often have associations with a broad range of neurodevelopmental and psychiatric phenotypes^27^. Similar findings have emerged from a sequencing study of ADHD, which highlighted strong overlap in the genes implicated in ADHD and ASD^3^, whereas GWAS demonstrate a moderate (*r*_g_ = 0.36) genetic correlation between ADHD and ASD^28^, and a previous CNV study suggested that the same biological pathways are impacted by CNVs in ADHD and ASD^29^. Our study adds to the growing body of literature supporting the relatively recent reconceptualization of ADHD as a neurodevelopmental disorder, as evidenced by its DSM-5 definition.

However although there is overlap in implicated CNV regions (see Table 2), the type of CNV is usually but not always the same. In particular, we observed a de novo 22q11 duplication and although such duplications have been observed to be associated with risk of other neurodevelopmental disorders, they are protective for schizophrenia^22^, whereas deletions at this locus (not observed here) are associated with schizophrenia as well as other neurodevelopmental impairments^21^.

One potential limitation of the study is that the proband DNA was more likely to come from saliva than blood (68.6% samples), whereas the parental DNA samples were more likely to come from blood (28.7% samples from saliva in mothers and 44.2% in fathers). Although the QC metrics were somewhat poorer for the saliva samples, given the careful QC protocol and examination of all the traces (which were definitive; see Supplement), the rate of de novo CNVs in this study is unlikely to be overstated. Collecting blood samples from children with severe neurodevelopmental disorders poses a challenge for genetic research studies. Furthermore, although the comparison of the results of this study to previously published studies is limited by such differences in source of DNA, different genotyping arrays and variable CNV-calling protocols, our focus on well-defined large, rare CNV loci is a robust approach that is unlikely to be biased towards detecting false positives, but may have resulted in missing valid de novo CNVs in the sample.

Although ADHD is highly heritable, complex and polygenic, rare mutation discovery has been slower than for many other similarly heritable and polygenic disorders that include DD/ID, schizophrenia, ASD and Tourette disorder. For example, de novo CNV data are currently only available for around a total of 500 trios (Lionel and colleagues^16^ and the current study) and there have been no large, trio-based sequencing studies of ADHD. To identify associated variants, as well as damaging and likely causal rare mutations, will require very large sample sizes of ADHD that are not yet globally available.

In summary, we present findings which suggest that de novo CNVs likely contribute to ADHD risk and highlight the need for larger discovery studies for future biological insights.

## Supplementary information

Supplementary Figure 1
