# Supplementary material for: A brief report: de novo copy number variants in children with attention deficit hyperactivity disorder
Source: Transl Psychiatry. 2020 May 12;10:135. doi: 10.1038/s41398-020-0821-y (PMC7217839; doi:10.1038/s41398-020-0821-y)

Supplementary Figure 1:
Probe intensity traces for *de novo* CNVs in each parent-offspring trio

A)

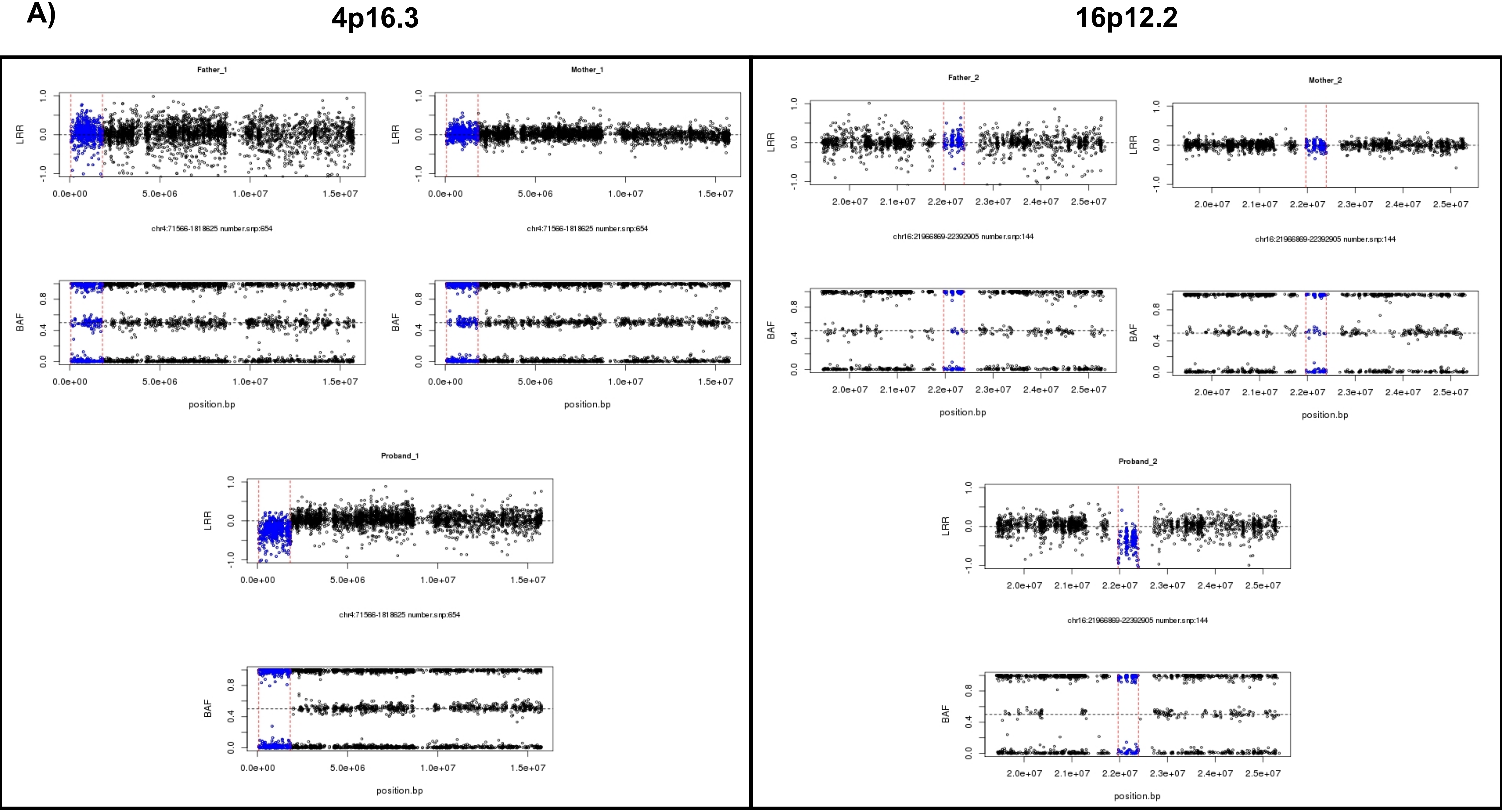

4p16.3
16p12.2
Father_1
Mother_1
Proband_1
chr4:71566-1818625 number.snp:654
Father_2
Mother_2
Proband_2
chr16:21966869-22392905 number.snp:144
LRR
BAF
position.bp

B)

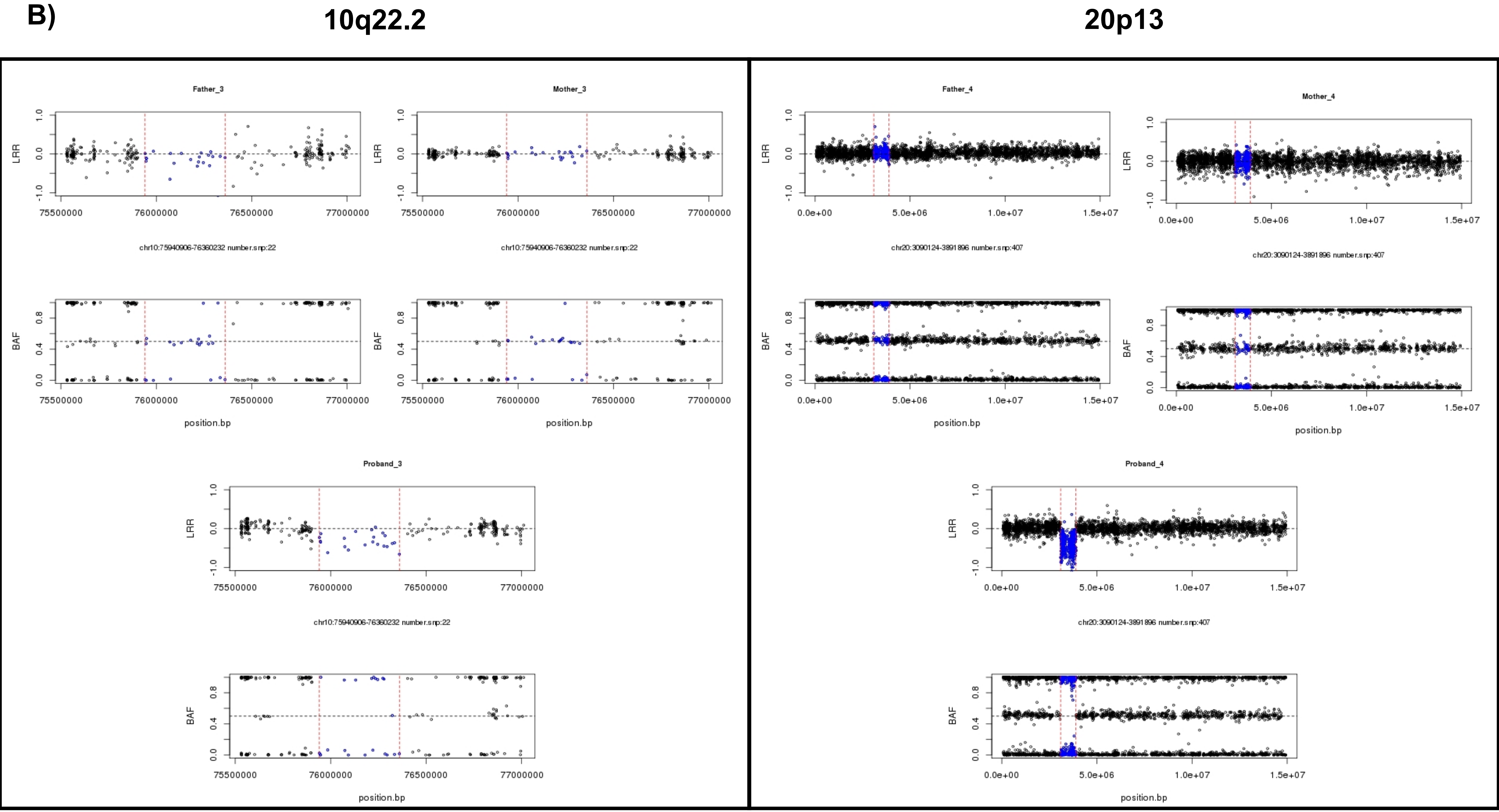
10q22.2
20p13
Father_3
Mother_3
Proband_3
Father_4
Mother_4
Proband_4
LRR
BAF
position.bp
chr10:75940906-76360232 number.snp:22
chr20:3090124-3891896 number.snp:407

C) 10q11.22-q11.23 15q26.3

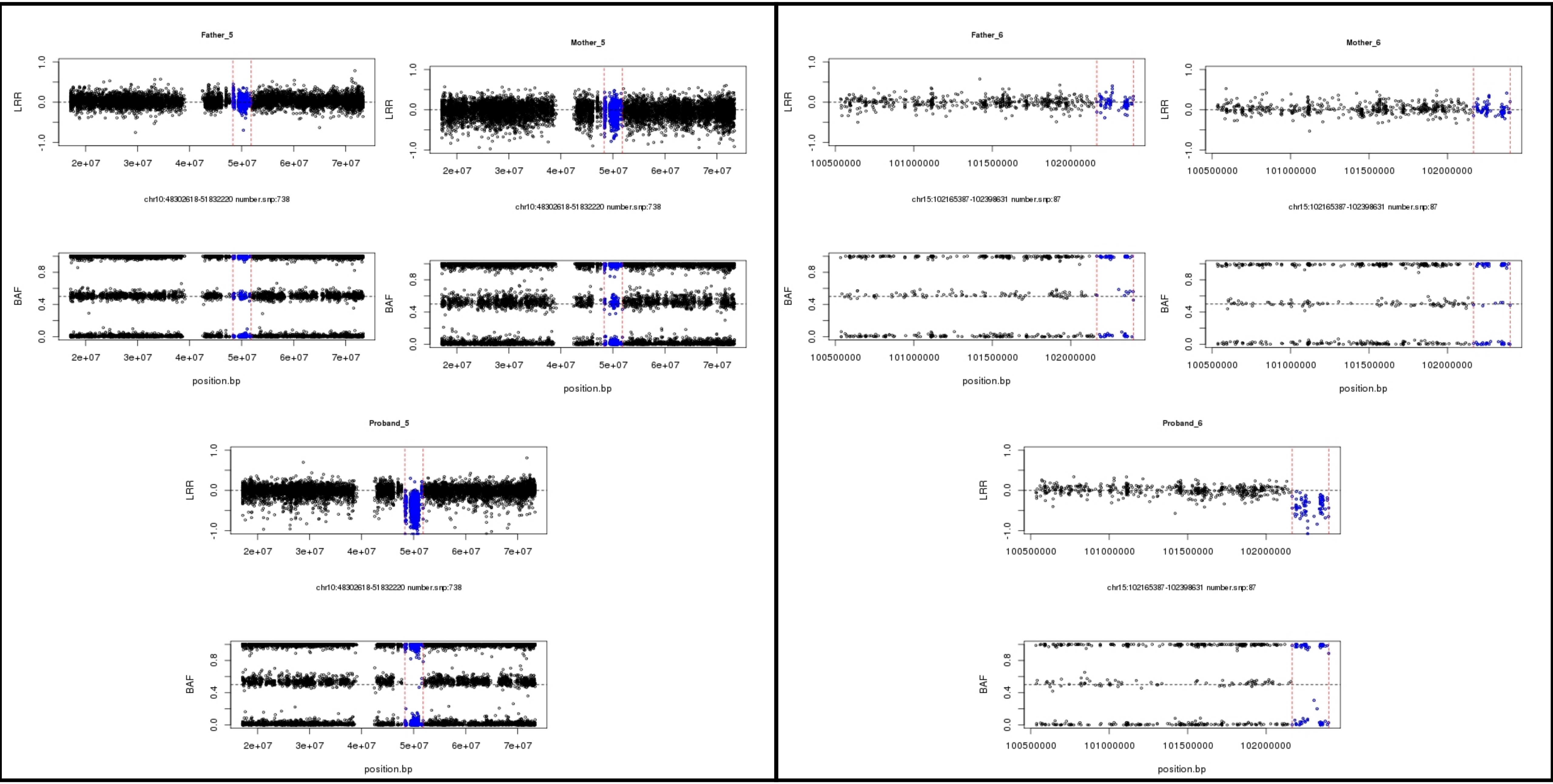

D)

15q13.1-q13.2

22q11.21

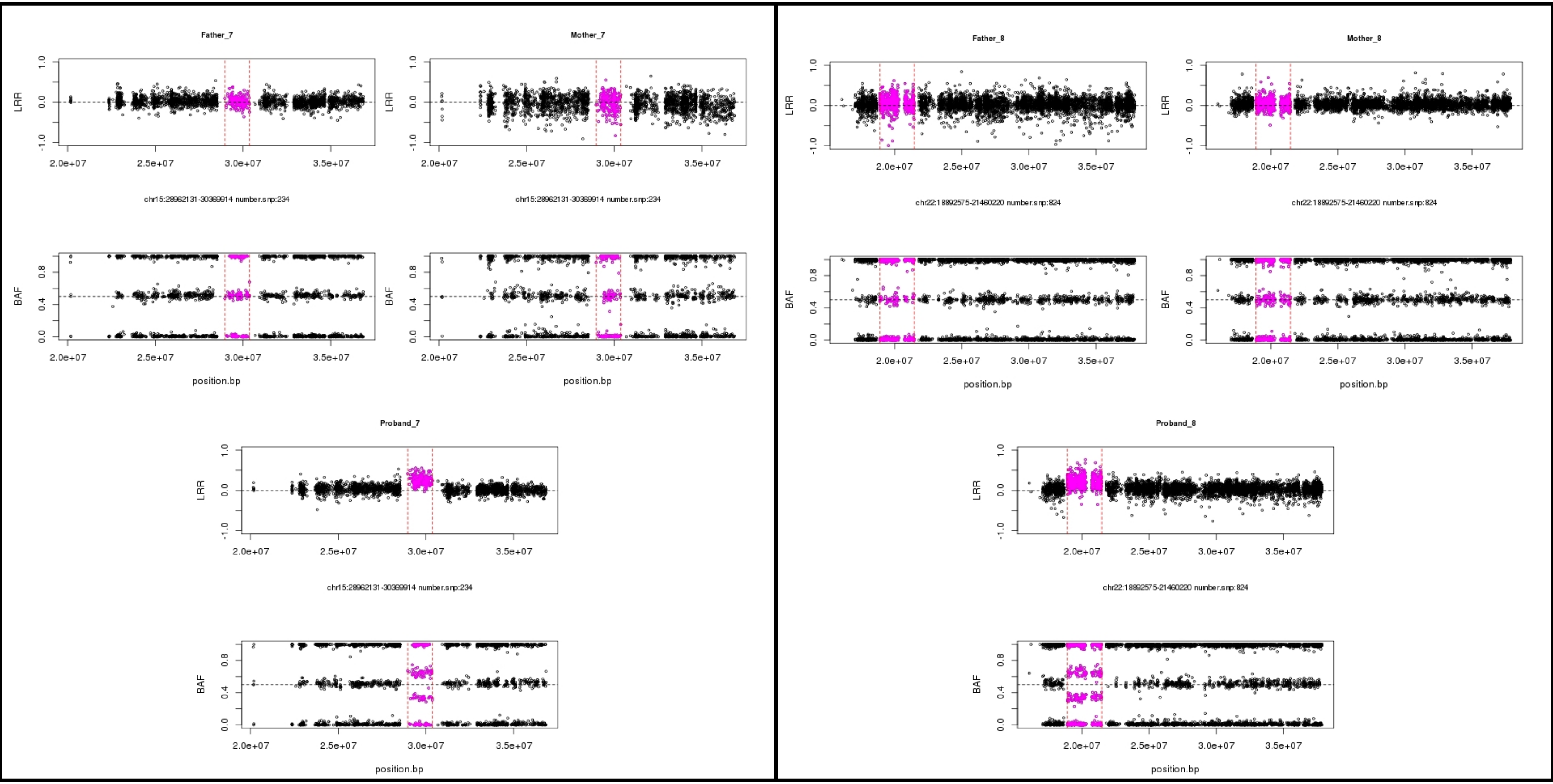

E) 4q13.1-13.2 4q22.1

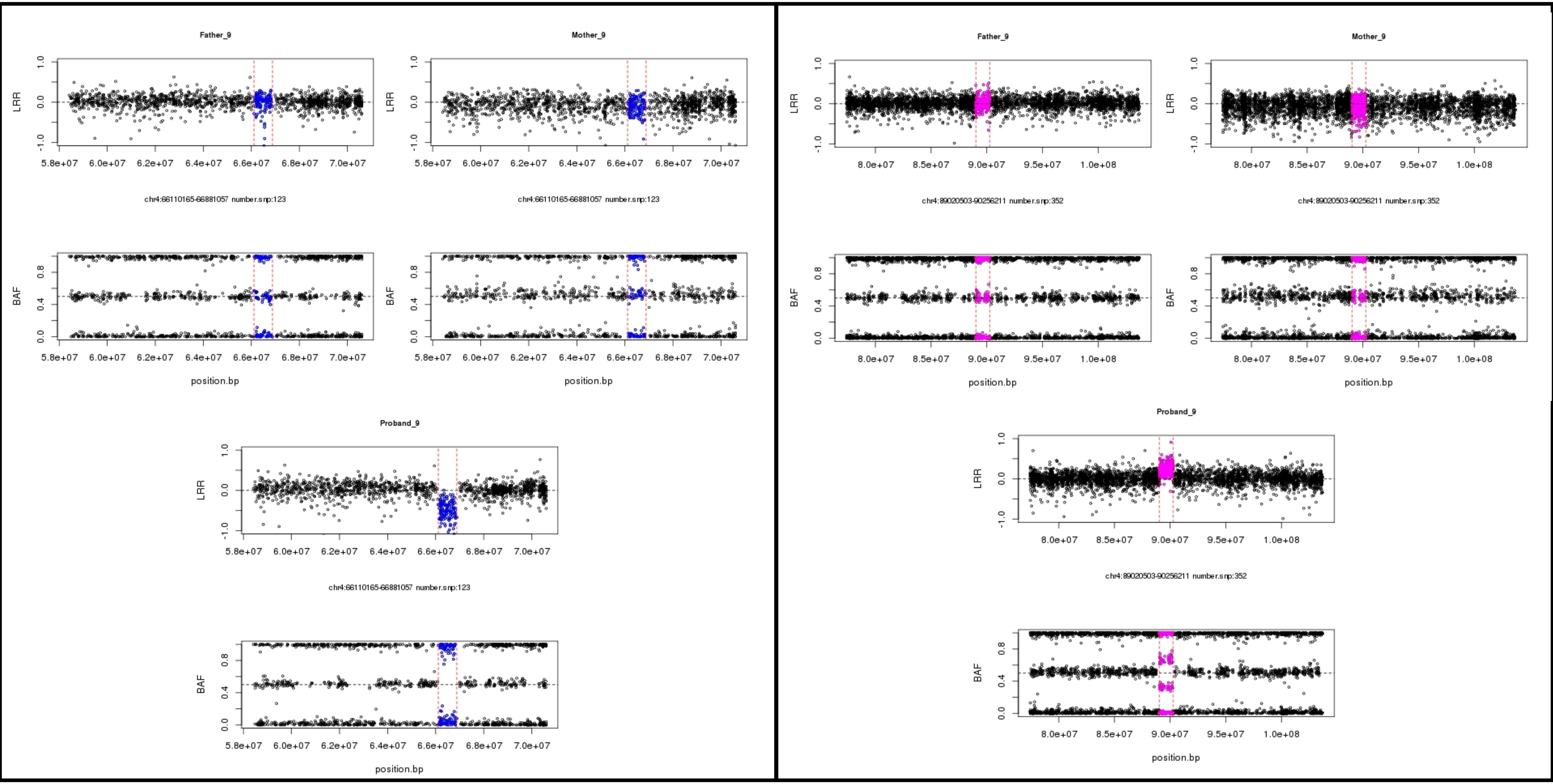

F)

16p13.3

16p13.11

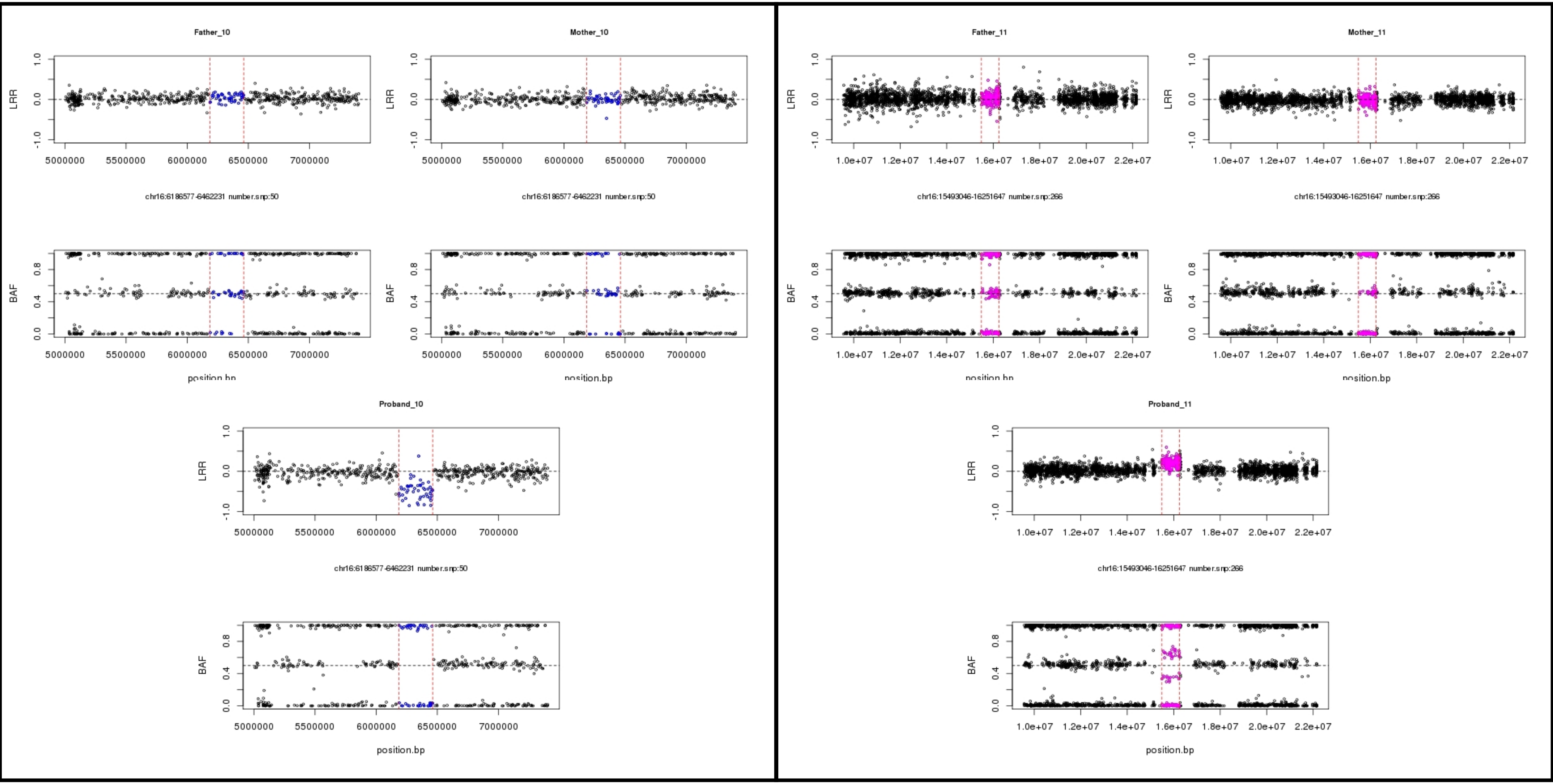

G)

11p15.4

10q21.3

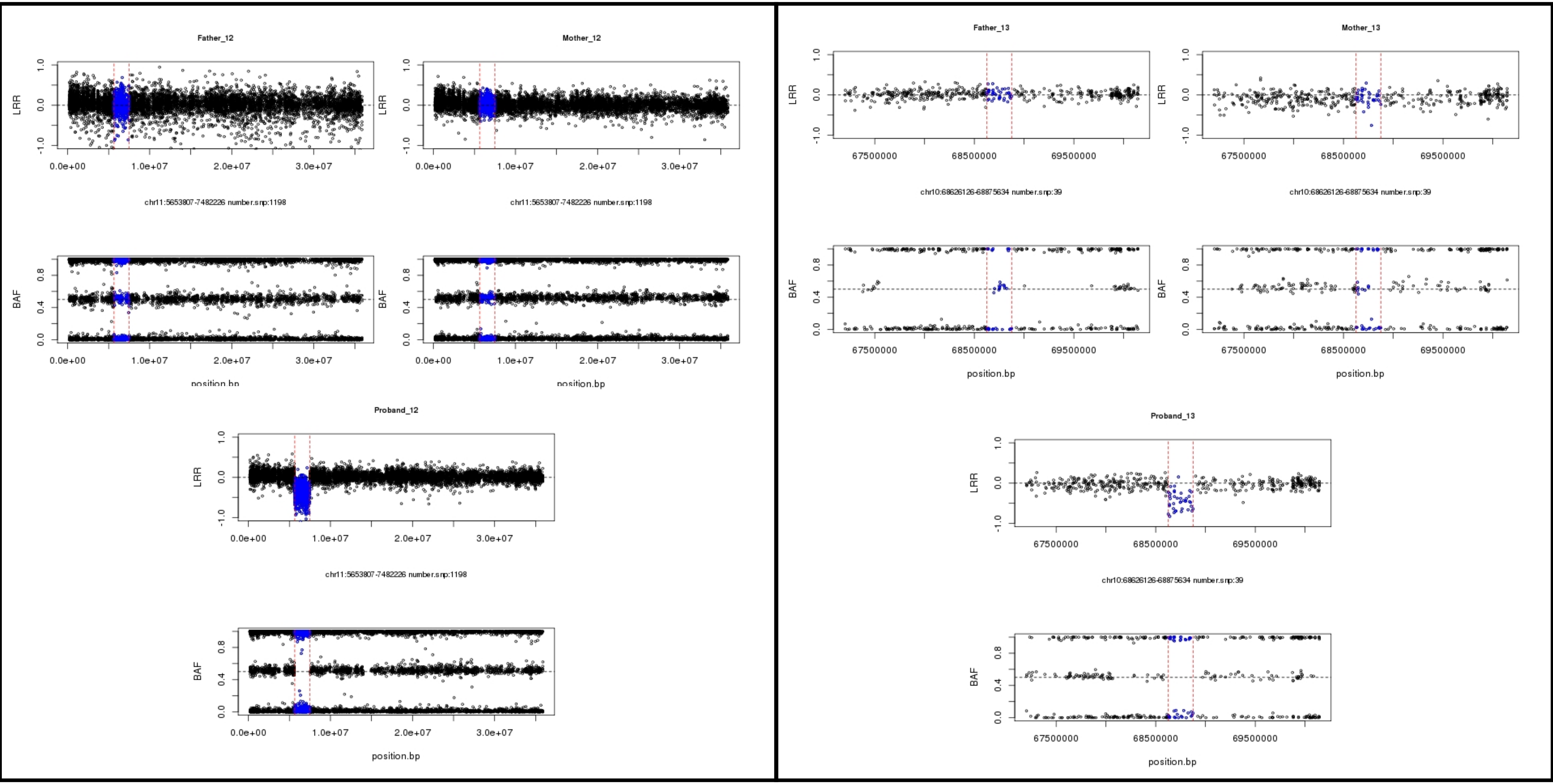

Supplement: Supplementary file 1 — Supplementary Figure 1 [file 41398_2020_821_MOESM1_ESM.pdf]
